# Steatite Powder Additives in Wood-Cement Drywall Particleboards

**DOI:** 10.3390/ma13214813

**Published:** 2020-10-29

**Authors:** Viet-Anh Vu, Alain Cloutier, Benoît Bissonnette, Pierre Blanchet, Christian Dagenais

**Affiliations:** 1 Department of Wood and Forest Sciences, Université Laval, Quebec, QC G1V 0A6, Canada; viet-anh.vu.1@ulaval.ca (V.-A.V.); pierre.blanchet@sbf.ulaval.ca (P.B.); christian.dagenais@fpinnovations.ca (C.D.); 2 Department of Civil and Water Engineering, Université Laval, Quebec, QC G1V 0A6, Canada; benoit.bissonnette@gci.ulaval.ca; 3 FPInnovations, Quebec, QC G1V 4C7, Canada

**Keywords:** steatite, wood particles, Portland cement, fire performance

## Abstract

The objective of this study was to develop a new drywall wood-based particleboard as an alternative to gypsum board. Various development iterations have led to the use of wood particles, steatite powder and Portland cement. The resulting outcome shows that screw withdrawal resistance was improved by 37% and bending properties by 69% compared to gypsum board of a similar density (0.68–0.70). The raw surface of the boards is of good quality and comparable to the paper-faced surface of gypsum board. Furthermore, the reaction to fire was evaluated through bench-scale test with a cone calorimeter. The investigated particleboard did not reveal visual signs of combustion after 20 min when exposed to a radiant heat of 50 kW/m^2^, while burning of the overlay paper of gypsum board occurred at about 57 s, suggesting that wood-cement-steatite powder particleboard could be classified as a quasi non-combustible material.

## 1. Introduction

Steatite (also known as soapstone or soap rock) is a type of metamorphic rock. It is primarily composed of mineral talc, rich in magnesium. Its main component is hydrated magnesium silicate:Mg_3_Si_4_O_10_(OH)_2_. As it is relatively soft because of its high talc content, it has been used as carving material for thousands of years. This stone is soft, dense, heat-resistant and has a high specific heat capacity [1]. Steatite can be pressed into complex shapes before heating. It is also used in the paint industry, particularly in marine paints and protective coatings for ceramics due to its high electrical resistivity [2]. Due to its electrical characteristics, steatite is mostly used in electrotechnics. In the world market, steatite with more than 92% brightness, less than 1.5% CaCO_3_ and less than 1% Fe_2_O_3_ is preferred for exports [3]. 

Many studies have been carried out to evaluate the performance and applications of wood-cement composites because of their low cost and important contribution in mitigating the housing problem in developing countries [4]. Indeed, many studies have shown that wood-cement boards, could be used for ceilings or walls covering [5,6]. The most important advantages of wood–cement boards are their high resistance to insect, fungi, decay, acoustic waves and fire [6,7]. In fact, the sugars present in wood can inhibit cement setting. Therefore, the main problem in wood-cement board design is the compatibility between wood and cement [6]. The effect of wood on cement setting depends on several factors, among which harvesting season and wood species have the higher impacts [8]. Several special cement-based mortar containing additions of fine powder such as steatite [9], glass [10] and wood ash [11] have emerged.

The replacement of cement with steatite powder (SP) decreases setting time of cement and increases mortar cube compressive strength, but the consistency of the binding material increases [2]. The replacement of cement with SP was reported to result in improvements of the mortar microstructure, up to maximum replacement rates of the order of 20% by weight [12]. 

Gypsum boards (GB) are widely used in North America building construction for interior partitioning. Gypsum boards consist of calcium sulphate in the form of dihydrate crystals with overlay paper on both sides. The board core is a non-combustible material. It contains nearly 21% chemically combined water which is slowly released as steam when submitted to high levels of heat. Because steam does not exceed 100 °C at normal atmospheric pressure, it effectively retards the transfer of heat and the spread of fire [13,14]. Even after complete calcination, when all the water has been released from its core, GB continue to serve as heat-insulating barriers. When installed in combination with other materials such as walls and ceiling assemblies, GB serve to protect building elements from fire effectively for prescribed durations. While GB fails the flaming criteria for determining the non-combustibility of materials due to the paper overlay [15], it is typically an accepted material for non-combustible construction in most building codes due to its good fire performance. However, the paper overlay plays a vital role in the mechanical resistance of GB [16]. Besides, it appears that construction wastes from this material are a problem [17], which is aggravated by its extensive use. Economic pressures and environmental concerns are some of the driving forces of today’s industrial development. Hence, many research projects are being conducted for increasing the utilization of waste materials in order to decrease threats to the environment and to streamline existing waste disposal and recycling methods by making them more affordable [17]. On the market, several alternatives to gypsum have been used such as plastic panels, plywood, fiberboard and veneer plaster.

The aim of the present study was to evaluate the mechanical, physical and thermal properties and reaction to fire of wood-cement particleboards incorporating SP as a supplementary cementing material, intended as an eco-responsible alternative to the GB. In this study, two in three of the raw materials used for particleboard production, wood particles and SP, are secondary low-cost products from lumber production and mineral extraction of steatite. 

## 2. Materials and Methods 

### 2.1. Material

The primary binder used was Portland cement type 10 (GU, General Use), an ordinary CSA (Canadian Standards Association).

The SP selected for this research project was provided by Polycore Inc, Quebec, Canada. 

The wood-cement mixtures were prepared with air-dried wood particles obtained from white spruce (*Picea glauca*) trees harvested at the Petawawa Research Forest in Mattawa (ON), Canada. The wood chips were refined with a Pallmann PSKM8-400 ring refiner (Ludwig Pallmann K.G, Zweibrücken, Germany). Then, the wood particles were screened using nine sieve sizes: 1.19, 1.40, 1.70, 2.38, 2.80, 3.35, 4.00, 4.46 and 5.00 mm.

The regular GB used in the study for comparison purposes were 12.7 mm [1/2 in] in thickness. They are commercialized by Georgia Pacific under the trade name ToughRock^®^. They were typical regular drywall boards used for interior partitioning in building construction.

### 2.2. Material Characterisation

#### 2.2.1. Wood Particles

**Figure 1:**
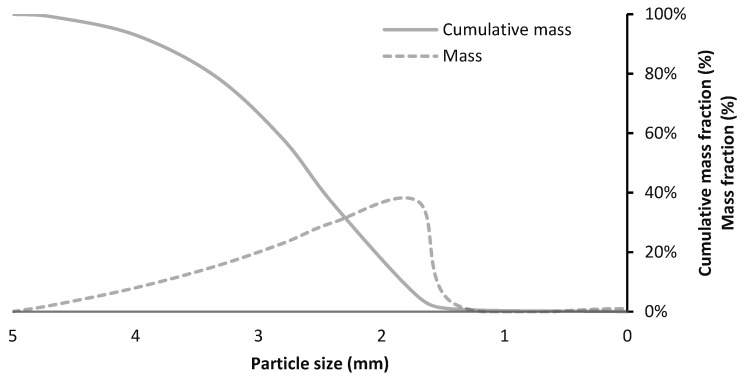
Wood particles size distribution.

Figure 1 shows the wood particles size distribution by mass. According to the results, all of the particles are smaller than 5 mm in size and the highest volume fraction (37%) is the particles with a diameter of 1.7 mm. In the study of Vu et al. [11], the size of the wood particles was less than 3 mm and the highest volume fraction was 1.7 mm. Wood particles size reaches a maximum of 5 mm for the purpose of increasing the mechanical strength of the particleboard.

#### 2.2.2. Steatite Powder 

##### Chemical Composition

Table 1 shows the results of the chemical analysis of SP. The combined content of aluminum oxide (Al_2_O_3_ = 0.7%), iron oxide (Fe_2_O_3_ = 6.32%), and silicon dioxide (SiO_2_ = 38.3%) reaches 45.32%, while the minimum value required for the material to qualify as a pozzolan is 70%. The relative mass loss during combustion observed at 950 °C was 20.4%, which is considerably more than the maximum requirement for pozzolans set at 12%. The alkali content recorded (%Na_2_O + 0.658 × %K_2_O) was less than 0.23%, which is lower than the maximum alkali content of 1.5% required for pozzolans [18]. Therefore, SP does not qualify as a pozzolan. The specific gravity of SP was found to be 2.91. This is lower than the specific gravity of Portland cement (3.15), but larger than for mineral aggregates typically used in cementitious materials (limestone, granite, quartzite). 

##### Particle Size Analysis

**Figure 2:**
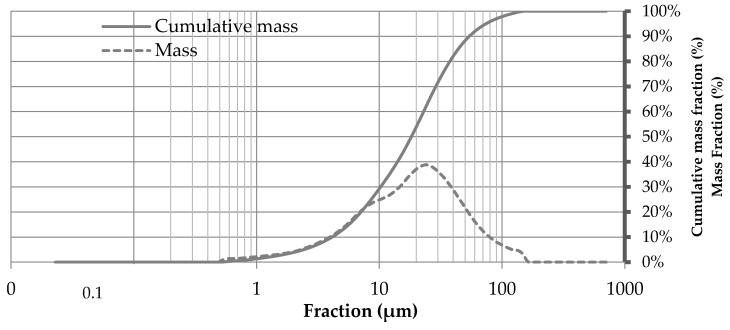
Particle size analysis of SP.

The most commonly used metrics when describing particles size distributions are D-Values (D10, D50 and D90) which are the intercepts for 10, 50 and 90% of the cumulative mass [19]. According to the results shown in Figure 2, D10, D50, and D90 values of the SP were 3.9 μm, 18.5 μm and 52.3 μm, respectively. The D90 value of the SP was smaller than the corresponding values (114.1 µm) recorded for wood ash in the study of Vu et al. [11]. Moreover, the tested material contained 14% of ultrafine particles (ϕ < 5 μm). Therefore, SP is suitable for use as a filler to reduce the porosity in the particleboard.

##### Material Preparation

**Figure 3:**
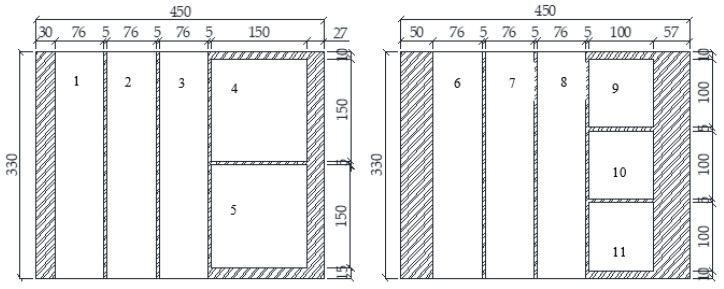
Sketch of samples cutting for WCSP (all measurements in mm).

The wood-cement steatite powder (WCSP) mixtures tested in this project were all prepared with the same ratio by weight of wood-binder and SP-binder, where the binder phase is the sum of cement and SP. The wood-binder ratio and SP-binder ratio selected were 0.35 and 0.15 (Table 3—P3). After mixing the materials in the mortar mixer, each particleboard was cast using the same 450 × 330 × 14 mm^3^ wooden mold. The wet mixture was poured into the mold, the surface was then levelled off with a wood screed, and in the end a wooden lid was secured on top of the mold with C-clamps. The particleboard thickness was reduced to 13 mm due to the pressure of the lid. The particleboards were unmoulded at the age of 3 days and stored in a conditioning chamber at 23 °C and 60% R.H. The various test specimens were sawn from the particleboard using a 5 mm thick saw blade at the age of 28 days (Figure 3). Particleboards nos. 1, 2, 3, 6, 7 and 8 were tested for bending modulus of rupture (MOR) and modulus of elasticity (MOE), and screw-withdrawal later. Thermal properties and water absorption tests were carried out on particleboards nos. 4 and 5. The reaction to fire was determined on particleboards nos. 9 and 10.

**Figure 4:**
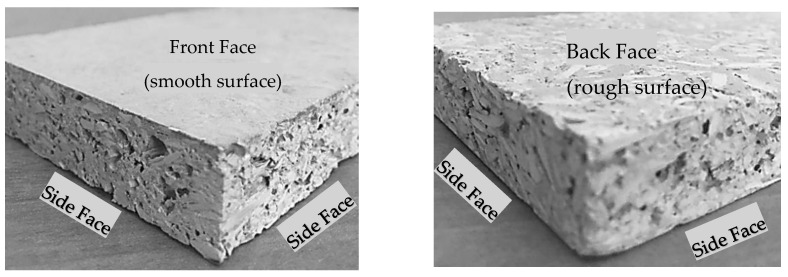
Edges and faces of a wood–cement steatite powder particleboard cut with a saw.

Due to the settling of the SP at the bottom of the panels, this face of the WSCP which was in contact with the mold had a less porous, denser microstructure than at the top. This face is the smoothest and is called front face. The top face of the panel in the mold which is the roughest is referred to as back face throughout this paper (Figure 4) and should be used against the structure when mounting a wall. In Section 2, the front face will be used for reaction to fire testing and nail pull resistance testing, while the three-point bending test is applied on both faces of the WCSPs.

### 2.3. Test Methods

In this study, the mechanical properties of the investigated particleboards and GB were determined in accordance with ASTM D1037-12 Standard test methods for evaluating the properties of wood-based fiber and particle panel materials [20]. Beside, the nail pull resistance test were determined in accordance with ASTM C473-17 Standard test methods for physical testing of gypsum panel products [21]. In both method, MOR and MOE, screw withdrawal resistance and nail pull resistance were determined using an MTS QTest-5 Universal Test Frame (MTS systems corporation, Eden Prairie, MN, USA) featuring the Elite Modular Control System. All experiments on WCSP test specimens were conducted at the age of 28 days. As shown in Figure 4, the molded WCSP samples have the shape of a panel. Therefore, the determination of density was based on the weight and the average dimensions of the samples.

Water absorption was determined in accordance with ASTM D1037-12. The reaction to fire was tested following the ISO 5660 [22] using a cone calorimeter (Fire testing technology Limited, West Sussex, UK). Thermal capacity, specific heat and thermal conductivity were determined with a FOX 314 Heat Flow Meter (TA instruments-LaserComp Inc., Wakefield, MA, USA) following the ASTM C518 [23]. The sample was placed between the two plates of the heat flow meter at a controlled temperature. The flux meter was attached on each side of sample. The temperature and heat flux could therefore be measured at the board surface. The bottom face of WSCP (in the mold) is the exposed face in the test. The bottom face was exposed directly to the heat flux and spark igniter. The four parameters (two temperatures and two heat fluxes) can then be used to calculate heat capacity and thermal conductivity of the sample.

Finally, solid samples were observed under a Scanning Electron Microscope in order to analyse its microstructure by the JEOL JSM-840A (JEOL USA Inc, Peabody, MA, USA) equipped with an energy dispersive X-ray analysis system (EDS). The specimens were placed on double-sides adhesive tape and coated with a thin alloy of Au-Pd. The operating conditions were set at 15 kV.

### 2.4. Preliminary Work

A preliminary test program was conducted to evaluate the effect of SP when used in partial replacement of cement in a mixture of wood particles and cement. Seven mixtures were investigated, the variable being the fraction of cement replaced by SP. The mixing sequence used with a mortar mixer (HOBART A-120, Hobart Canada Inc, Don Mills, ON, Canada) is presented in Table 2.

Unsurprisingly, the presence of steatite was found to increase the amount of water necessary to produce mixtures with adequate workability. The quantity of water required was estimated according to ASTM C1437 [24] to make sure that all mixture have the same workability value as P1 (Table 3). Assessing the workability and bending strength of mixtures with different percentages of SP was intended to determine the maximum amount of SP that could be used in the mixture without affecting negatively the mechanical properties of the particleboard in comparison with those of the reference wood-cement particleboard and GB. Only cement and wood particles were selected to prepare the control mixture (P1), while six other mixtures were prepared by incorporating SP at replacement rates of 10, 15, 20, 30, 40 and 50% respectively (P2 to P7). 

Preliminary mechanical results have shown that the replacement of cement by SP in WCSP has a significant impact. The three-point bending test results at 3, 7, 14 and 28 days of moist curing show that the bending strength of the sample particleboards increases with the curing time as expected for Portland cement-based systems, although it does not increase much beyond the age of 14 days. A density change test revealed that the weight of all particleboards was stable after 14 days of curing. The study of Vu et al. [8] has also shown that the difference of bending resistance between the particleboard cement-wood-wood ash at 7 and 28 days of curing time, was not significant (4.2% max.). In the freshly consolidated particleboard, the heavier SP particles tend to settle in the bottom, yielding non-uniform characteristics across the thickness of the board. This segregation results in non-isotropic particleboards with different bending MOR depending on which side is subjected to tension stress during the test. These preliminary results have shown that particleboards with 15% of the cement replaced by SP (P3) is optimum, with the best mechanical properties obtained among the six tested mixtures. Indeed, the study of P. Kumar et al. [12] shown that the replacement of SP should be maintained below 20%.

## 3. Results

### 3.1. Change in Density

The variation of particleboard density was determined by the recording of weight of all particleboard at the begin and at the end of the curing period in the wood mold (from 0 to 3 days). A reduction of weight of about 5% during that period occurred due to water evaporation through the mold. After removal from the mold, the particleboard mass typically reached a plateau at about 6 days, meaning that most of the free water in the mortar had evaporated in the conditioning chamber at 23 °C and 60% R.H by then. At 14 days, the particleboards had a specific gravity ranging between 0.68 and 0.70.

### 3.2. Scanning Electron Microscopy

**Figure 5:**
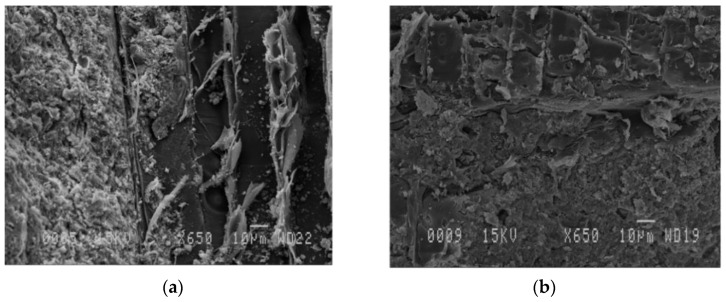
Scanning electron microscope images of cement-wood particles (**a**) and cement +15% replacement of powder steatite+ wood particles (**b**).

According to the results shown in Figure 5, both materials show a low porosity and pore sizes smaller than 10 µm. Based on SEM examination, the difference of microstructure of a mixture of neat cement-wood and a mixture containing 15% of SP in replacement of cement is not significant. Both materials show a uniform and dense microstructure.

### 3.3. Bending Properties

**Figure 6:**
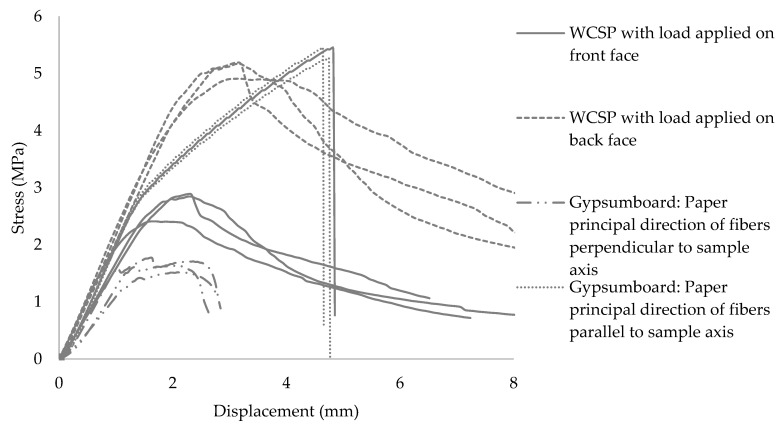
Characteristic stress–displacement curve for a three-point bending test of WCSP and GB in accordance with ASTM D1037-12.

Figure 6 presents the evaluation of bending properties obtained for WCSP and GB. Three replicates per products were tested. The average values of elastic modulus and bending strength for each tested specimen are shown in Table 4. 

As mentioned previously, 28 days after casting, the particleboards were tested in static bending using in each case six specimens. Separate test series were carried out with the front or back face subjected to bending test. Typical load-deflection curves obtained for these different test configurations are displayed in Figure 6. WCSP were tested with the load being applied on the front or on the back face of the samples. As explained before, due to the settling of the SP, the mortar at the front face of the WCSP has a less porous, denser microstructure than at the back face. Therefore, the bending strength when the load is applied on the front face of the sample is lower than on the back face of the WCSP. The WCSP with the load on the back face exhibited a bending strength of 5.1 MPa, which is over 1.9 times more than it is on the front face. The analysis of the stress–displacement curves indicates that there are three material behavior stages in the course of the test that corresponds to the mechanical behavior in the constituent composite materials (wood-cement-steatite). Each experimental curve included a linear period at the beginning of the test and a non-linear period later. The linear period represents the elastic behavior of the material. The tangent elastoplastic modulus decreases in the second period, which corresponds to a non-linear plastic behavior, the third period corresponds to the last part of the curve when the WCSP began to fracture. The MOE of WCSP is about 1.7 GPa with the load applied on the front face and about 2.1 GPa with the load applied on the back face.

For the GB, separate bending tests were conducted with the samples oriented perpendicular and along the direction of the overlay paper fibers (Figure 6). The respective stress–displacement curves in bending were entirely different. In the overlay paper fiber direction, the GB exhibit a fragile behavior, while it is more ductile in the perpendicular direction. This is due to the different tensile properties of the overlay paper in the two orthogonal directions. Therefore, for samples oriented perpendicular to the paper fiber direction, the paper failed at the beginning of the test and did not have a significant effect on the bending test. For samples oriented in the paper fiber direction, the overlay paper had a significant contribution to the mechanical properties and played the role of a reinforcement. Table 4 shows that the MOR in the overlay paper fiber direction is 5.4 MPa, which is 3.4 times higher than in the perpendicular direction. It is approximately equal to the MOR of WCSP in the case of a load applied on the back face and two times higher than in the case of a load applied on the front face. In fact, the mechanical quality of the GB depends significantly on the gypsum core properties. Therefore, the whole GB failed as the reinforcement failed. That is why the behavior is fragile (Figure 6). This mechanical comportment of GB was also noticed in the study of P. Tittelein et al. [6]. The GB bending MOE in the overlay paper fiber direction is 1.9 GPa, which is 1.5 times higher than it is in the perpendicular direction, 1.1 times lower than the bending MOE of WCSP in the case of loading on the back face and 1.1 times higher than it is in the case of loading on the front face. The results reveal that the MOR and MOE of WCSP in the case of loading on the front face are lower than in the GB overlay paper fiber direction and higher than across the GB overlay paper direction. However, the WCSP still could replace GB when adjusting the distance of the studs in the wall composition. A good bending strength of WCSP in the case of loading on the back face is an advantage for transportation and installation.

### 3.4. Screw Withdrawal and Nail Pull Test 

The results of the screw withdrawal and nail pull resistance tests are shown in Table 5. According to these results, WCSP has better resistance to screw and nail withdrawal than GB. The recorded screw withdrawal resistance and nail pull resistance of WCSP are respectively 37 and 11% higher than the corresponding values recorded for the GB. The resistance values of GB show less variation, as it is a more homogeneous material than WCSP.

### 3.5. Water Absorption

The water absorption test results are shown in Table 6. While the data show that water absorption of WCSP is just slightly lower than that of GB, the difference with respect to swelling is considerable. In fact, WCSP shows virtually no swelling, whereas for almost the same water uptake, GB undergoes a 5% expansion. In the case of WCSP, most of the free water in the mortar had evaporated about 6 days after removal from the mold based on the samples weight. Therefore, the cement was not fully cured leading to shrinkage in water. In addition, the negative value may be caused by the erosion of the specimen due to the flow of water during the test.

### 3.6. Thermal Properties

Table 7 shows the results obtained for thermal capacity, specific heat, and thermal conductivity of WCSP and GB using the test method described in Section 2.2. It is worth noting that WCSP has a thermal conductivity almost three times lower than that of GB. This low thermal conductivity results from the high porosity of the WCSP compared to GB. The thermal capacity and specific heat of WCSP is 1.4 times higher than that of GB. This indicates that WCSP’s ability to store thermal energy is higher than for GB. This is an important characteristic for building applications, namely those where fire-resistance rated components are required. 

### 3.7. Reaction to Fire

**Figure 7:**
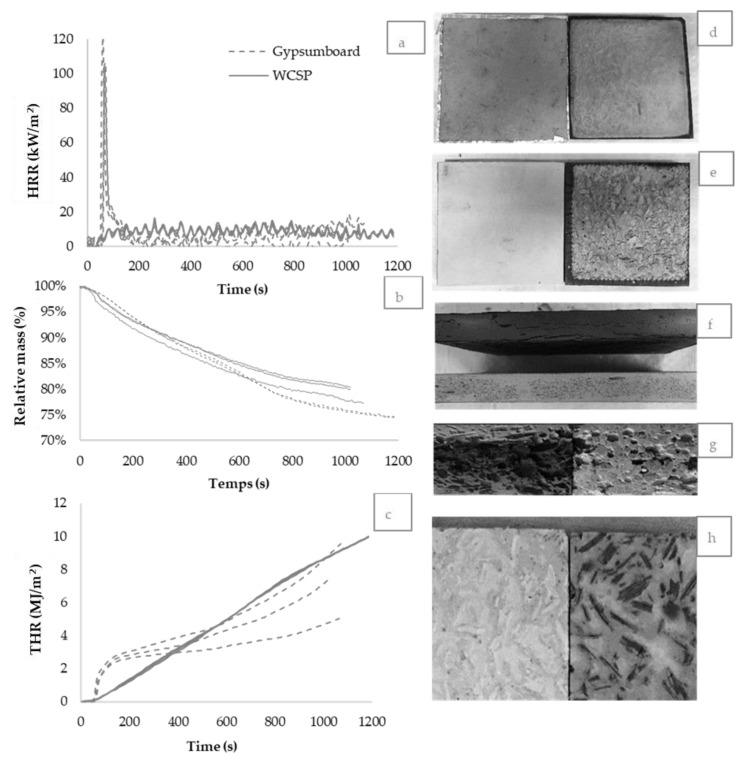
Cone calorimeter measurements: (**a**) Heat release rate (HRR). (**b**) Relative mass. (**c**) Total heat release (THR). (**d**) Front face of WCSP before (**left**) and after (**right**) testing. (**e**) Front face of GB before (**left**) and after (**right**) testing. (**f**) Side face of gypsum specimen before and after testing. (**g**) Side face of WCSP before (**left**) and after (**right**) testing. (**h**) Back face (**left**) before and after testing (**right**) of WCSP.

The thermal properties and results of the cone calorimeter tests are presented in Table 8 and Figure 7.

The WCSP with a SP replacement rate of 15% was tested for comparison with GB. Test results in Figure 7a indicate that the heat release rate (HRR) of GB increases very quickly and reaches a maximum at about 65 s, due to burning of the overlay paper occurring at about 57 s (Table 8). The WCSP showed very low HRR throughout the 20 min duration. The peak of heat release is not obvious as no combustion took place (Table 8). The HRR of WCSP varied from −2 to 16 kW/m^2^ and also varied from −4 to 12 kW/m^2^ in the case of GB after the ignition. Such variations are related to the accuracy of the cone calorimeter for materials exhibiting low combustibility characteristics, such as a THR less than 15 MJ/m^2^.

The National Building Code of Canada (NBCC) states that a material can be used in non-combustible construction provided that, when tested in accordance with CAN/ULC-S135 [25] at a heat flux of 50 kW/m^2^, the total heat release is no more than 3 MJ/m^2^ and the total smoke extinction area is no more than 1.0 m^2^.

Figure 7b shows that WCSP behaves similarly to GB with respect to mass loss. Indeed, the remaining mass of GB after 15 min is 81% while the WCSP is 78%. The average total smoke production for 15 min recorded for both materials are less than 1 m^2^ (Table 8). Their average total heat release (THR) exceed in each case 3 MJ/m^2^ (Figure 7 and Table 8). While CAN/ULC-S135 slightly differs from ISO 5660, the results suggest that WCSP would most likely fail these requirements due to its average THR exceeding 3 MJ/m^2^. Given the low value threshold of 3 MJ/m^2^, very few products consisting in whole or in part of combustible materials will pass this test [26]. It is unclear from the NBCC as to how this threshold value was determined.

In Japan, based on the performance during a cone calorimeter test when subjected to an irradiance level of 50 kW/m^2^, the reaction to fire of interior finishing materials are classified as being non-combustible, quasi-non-combustible or fire retardant [27]. Neither WCSP nor GB actually meet the Japanese criteria of a non-combustible material: THR ≤ 8 MJ/m^2^ and peak rate of heat release ≤200 kW/m^2^ after 20 min of exposure. It is noted that the Japanese THR threshold of 8 MJ/m^2^ is much less severe than the Canadian value of 3 MJ/m^2^. They would however both meet the criteria for a quasi-non-combustible material: THR ≤ 8 MJ/m^2^ and peak rate of heat release ≤200 kW/m^2^ after 10 min of exposure. 

Flame-spread, which is used to describe the surface burning characteristics of building materials, is one of the most commonly tested fire performance characteristics for limiting fire growth in the early stage of fire development. The results generated in this study show that the surface of WCSP is still not burnt after 20 min. The surface color barely changed and neither fractures nor burnt surface could be observed in Figure 7d). The side face of the specimen became dark due to direct contact with the metallic specimen holder and the aluminum wrap, while the surface of the GB burned completely (Figure 7e). The side face of the sample shows that the GB burned and became dark (Figure 7f) across the thickness, while the 3 mm surface layer of the WCSP almost did not change color (Figure 7g) and did not burn. However, the wood particles on the inside and back face of the WCSP became dark due to the high temperature and large porosity (Figure 7g,h). It may explain why the HRR of WCSP (−2 to 16 kW/m^2^) was slightly higher than that of GB (−4 to 12 kW/m^2^) after the end of ignition of GB (Figure 7a).

## 4. Conclusions

Due to the settling of steatite powder, the formed surface (bottom face) of a wood-cement steatite powder (WCSP) board was of good quality even without paper overlay. It compares favorably to the surface of paper-faced gypsum boards. Besides, the ASTM D 1037-12 screw withdrawal resistance and ASTM C473-15 nail pull resistance of wood-cement-steatite powder boards were found to be 37% and 11% higher, respectively. When the load was applied on the front face, their bending strength is 69% higher. These panels also exhibit better water-resistance and better reaction to fire than those of gypsum boards. Indeed, with regards to reaction to fire, no ignition was observed for the WSCP, and the remaining mass of both type of boards after 15 min from start of the test was similar. The test results obtained in the present study actually show that wood-cement-steatite powder boards could be classified as a quasi-non-combustible material. While the replacement of cement with steatite powder at a rate of 15% improved the mechanical and thermal properties of the panel, it could also contribute to reduce CO_2_ emissions caused by cement production. Two-thirds of the raw materials used for wood-cement-steatite powder board production are low cost secondary products from mineral extraction of steatite and lumber production. The above results show that replacing gypsum boards by such an engineered material may be a worthy choice for buildings of the future.

## Figures and Tables

**Table 1:** Composition and properties of steatite powder.

Chemical Composition (%)		Property	Value
SiO_2_	38.3	Density	2.91 g/cm^3^
Al_2_O_3_	0.70	Blaine fineness	6505 cm^2^/g
Fe_2_O_3_	6.32	pH	9.4
MgO	33.9		
CaO	0.77		
Na_2_O	0.22		
K_2_O	<0.01		
TiO_2_	0.02		
MnO	0.09		
P_2_O_5_	<0.01		
Cr_2_O_3_	0.34		
V_2_O_5_	<0.01		
ZrO_2_	<0.02		
ZnO	<0.01		
PAF	20.4		

**Table 2:** Mixing Sequence.

Step	Mixer Rotor Speed (rpm)	Cumulative Time (s)
Addition of cement and wood ashes	140	0
Addition of water	140	60
Addition of wood particles	140	120
	285	180
End of mixing	0	270

**Table 3:** Mass ratio of steatite powder, cement and water used for the seven mixtures considered.

Mass Ratio	P1	P2	P3	P4	P5	P6	P7
Steatite powder/Cement	0.00	0.10	0.15	0.20	0.30	0.40	0.50
Wood/(Steatite powder + cement)	0.35	0.35	0.35	0.35	0.35	0.35	0.35
Water/(Steatite powder + cement)	1.00	1.15	1.24	1.32	1.45	1.56	1.65

**Table 4:** Bending strength of WCSP and GB (s = standard deviation) accordance with ASTM D1037-12.

Property		GB	WCSP
Specific gravity		0.7 (s = 0.02)	0.68 (s = 0.2)
Sample parallel to paper fiber direction	MOR (MPa)	5.4 (s = 0.08)	
	MOE (GPa)	1.9 (s = 0.03)	
Sample perpendicular to paper fiber direction	MOR (MPa)	1.6 (s = 0.08)	
	MOE (GPa)	1.3 (s = 0.04)	
Load on front face	MOR (MPa)		2.7 (s = 0.2)
	MOE (GPa)		1.7 (s = 0.24)
Load on back face	MOR (MPa)		5.1 (s = 0.12)
	MOE (GPa)		2.1 (s = 0.09)

**Table 5:** Screw withdrawal and nail pull resistance of WCSP and GB (s = standard deviation).

PROPERTY		GB	WCSP
Screw withdrawal resistance test (N)	ASTM C473-15	374 (s = 8)	415 (s = 20)
Nails pull resistance (N)	ASTM 1037-12	328 (s = 7)	450 (s = 38)

**Table 6:** Moisture absorption characteristics of WSCP and GB according to ASTM D1037-12.

Property	GB	WCSP
Water absorption (%)	54	51
Thickness swelling (%)	5	−1

**Table 7:** Thermal properties of WCSP and GB according to ASTM C1784-13.

Property	GB	WCSP
Thermal capacity (kJ/kg·K)	970	1338
Specific heat (kJ/m^3^·K)	679	910
Thermal conductivity (W/m·K)	0.32	0.12

**Table 8:** Results of the test calorimeter cone for WCSP and GB in accordance with ASTM E1354-17 (from start of test +15 min).

Property/Characteristic	GB	WCSP
Average ignition time (s)	57	none
Average peak rate of heat release (kW/m^2^) for 15 min	109.87	15.03
Average time to peak rate of heat release (s)	65	423
Average total smoke production (m^2^) for 15 min	0.19	0.28
Average total heat release (MJ/m^2^) for 15 min	5.89	7.88
